# Analysis of the Correlation Between the Governance and Quality of Biomedical Waste Management in Public Health Facilities in Togo, 2024

**DOI:** 10.3390/ijerph22071089

**Published:** 2025-07-08

**Authors:** Sarakawa Abalo Niman, Edem Komi Koledzi, Nitale M’balikine Krou

**Affiliations:** 1 Laboratory of Waste Management, Treatment and Recovery (GTVD), Faculty of Sciences, University of Lomé, Lomé BP 1515, Togo; edemledzi@yahoo.fr; 2 Regional Excellence Centre on Sustainable Cities in Africa (CERViDA-DOUNEDON), University of Lomé, Lomé BP 1515, Togo; 3 Laboratory of Organic Chemistry and Environmental Sciences (LaCOSE), Faculty of Science and Technology, University of Kara, Kara BP 404, Togo; krounitale@gmail.com

**Keywords:** governance, quality, management, biomedical waste, public health facilities, Togo

## Abstract

Increasing the use of healthcare facilities has resulted in the growing production of biomedical waste, which poses health risks to users, health professionals, and the environment. The aim of this research is to study the correlation between governance in Togo’s public health facilities and the quality of biomedical waste management within these facilities. Methods: This was a cross-sectional, descriptive, and analytical study conducted from September to December 2024. It involved 264 public health facilities of all types in all health regions of Togo. Health facilities were selected using the simple random selection technique. Healthcare providers were selected using the reasoned choice technique. The statistical tests used were the chi-square test and logistic regression, which enabled proportions to be compared and confounding factors to be eliminated, respectively. Results: Multivariate analysis revealed a statistically significant association between the organization and training component of governance and the quality of biomedical waste management (BMWM) in health facilities (OR = 3.79; 95% CI [1.79–8.03]; *p* < 0.001). This relationship suggests that health facilities with functional infection prevention and control (ICP) or BMWM committees, trained staff at all levels (nursing, technical, and administrative), and dedicated waste management personnel are more likely to implement compliant waste management practices. Analyses of the data also revealed that, among the criteria for assessing the quality of biomedical waste management (BMWM), the most significant were sorting (OR = 1.482; 95% CI [1.286; 1.708]), quantification (OR = 2.026; 95% CI [1.491; 2.753]), transportation (OR = 1.403; 95% CI [1.187; 1.66]), and disposal infrastructure (OR = 1.604; 95% CI [1.298; 1.982]). The application of this grid shows that 17.8% of the health facilities surveyed had a score equal to or above 80% on all the criteria used to assess the quality of biomedical waste management, and they were therefore managing waste in an “acceptable” manner. The study highlights key findings in biomedical waste management practices, providing actionable insights for improving public health safety.

## 1. Introduction

The mission of any health training is to ensure the promotion of health through education of populations in health-friendly behaviors and the provision of quality care and services to populations. However, it must be noted that health facilities are places where all kinds of waste can be stored and are often managed inadequately. With serious implications in terms of care-associated infections, the spread of diseases with epidemiological potential and environmental pollution [1,2]. This situation requires special attention not only to protect the environment from the risks associated with these wastes but also to reduce their impact on the health of users [3].

In Togo, biomedical waste management (BMWM) is a major public health problem that has not yet been adequately addressed. Despite the adoption of successive regulatory frameworks and strategic plans, operational implementation at the level of health facilities still needs to be improved. According to the evaluation report of the National Strategic Plan [4], only 55% of health facilities are used for waste sorting, and anatomical or sharp waste is sometimes found in intermediate or final landfills, exposing children and waste pickers to direct health risks [5]. At the same time, less than 60% of health facilities have an incinerator, some of which is either not working or misused [4]. This poor performance in waste management is due to several factors, including the low level of awareness of health and environmental risks associated with the poor management of biomedical waste, insufficient initial and continuing training of health workers, inadequate biomedical waste management, insufficient supervision and internal control, poor regulatory adequacy and enforcement, and the absence of institutional incentives or sanctions [4].

This situation in Togo reflects a wider problem in sub-Saharan Africa. This deficiency is observed at all stages of the biomedical waste management chain, with a high level of risk of infection associated with the handling of sharps waste.

Indeed, regarding the risk of infection, the poor management of Sharp cutting and edging objects represents a major source of infection risks, particularly blood exposure accidents (HEP) caused by sharp and contaminated objects. A WHO survey in 22 low-income countries found that between 18% and 64% of health facilities do not have proper methods for the disposal of biomedical waste [2,6]. This management deficit has serious consequences. Indeed, the inappropriate management of waste from care activities could lead, among other things, to Blood Exposure Accidents (BEA) for healthcare providers as well as users, staff managing these wastes, local populations, and the contamination of water, soil, and air [6,7,8,9,10,11,12]. In 2010, 1.7 million new cases of hepatitis B, 315,000 cases of hepatitis C, and 33,800 cases of HIV were attributed to blood exposure accidents related to prickly, sharp, and cutting medical objects from poorly managed biomedical waste [13,14]. This situation is related to the failure of several components of governance, notably the availability and application of procedures, the lack of training for healthcare providers and other stakeholders involved in biomedical waste management and the availability of equipment for the proper and safe sorting and transport of biomedical waste.

Regarding regional gaps in regulation, several studies conducted in West African cities (Bamako, Ouagadougou, Cotonou, Dakar) confirm that regulatory frameworks exist but suffer from a lack of operationalization, funding, and training [15,16,17,18]. This regulatory and institutional gap at the sub-regional and national level has implications at the health facility level, with poor consideration of biomedical waste management in the governance of health facilities. The marginalization of waste management in health facilities is reinforced due to the lack of consideration of waste management indicators in hospital performance at national level.

In Togo, despite the support of partners such as WHO, the World Bank or UNICEF, structural challenges remain in waste management, including the lack of systematic sorting, lack of appropriate equipment, insufficient training of staff, and low level of functionality of IPC/BMWM committees.

These findings highlight systemic deficiencies that directly refer to governance weaknesses in biomedical waste management [3,18,19,20]. Indeed, governance is understood here as a set of interrelated functions of regulation, planning and financing, organization and training. Each of these components plays a central role in activating the mechanisms necessary for effective management of biomedical waste. Indeed, the regulation allows the definition and implementation of standards, rules, and management procedures adapted to national and international standards. Planning and funding facilitate the integration of waste management interventions into overall health training budgeting, thus facilitating the financing of planned activities. The organization through the establishment of IPC/BMWM committees, training and oversight ensures that the procedures described by the regulatory component are properly applied for a sustainable improvement in the quality of waste management.

Thus, this work is based on a conceptual framework in which governance structures directly influence operational practices such as the sorting, quantification, transport, and disposal of biomedical waste. These practices in turn condition the quality of ecological and safe management of biomedical waste. This logic model establishes a causal relationship between governance and management quality.

Few studies in the African context, and none at this scale in Togo, have systematically explored this link between institutional governance and concrete results in biomedical waste management. Existing research often documents technical malfunctions and non-conformities in biomedical waste management without analyzing the underlying structural factors [21]. It is precisely this analysis that this study aims for, through examination of the influence of different components of governance on the quality of biomedical waste management in 264 public health facilities distributed in the country’s six health regions.

By highlighting the links between governance and performance in waste management, this study aims to make an original contribution to the improved targeting of levers and actors for the sustainable improvement of biomedical waste management. In particular, it identifies short-term governance levers such as staff training, the functionality of waste management committees, and the integration of BMWM into action plans to sustainably strengthen health and environmental security in Togo’s public health facilities.

We assume that health establishments with better governance, particularly in terms of organization and training, have a better quality of biomedical waste management.

## 2. Study Methods

### 2.1. Framework of Study

This research study was carried out in public health establishments of all types (HU type I, HU type II, district hospital type I, district hospital type II, regional hospital centers, and university hospital centers), and it included all six regions and thirty-nine health districts of Togo.

The organization of the health system is pyramidal and has three levels: central, regional, and operational. The structures and responsibility for waste management are also structured according to this pyramid organization.

Thus, at the central level, the Public Health Directorate, the Hygiene and Sanitation Directorate in collaboration with the Sanitary Infrastructure Directorate, Equipment and Maintenance, and the Management of Care Establishments have the task of developing guidelines in accordance with international standards, resource mobilization and development of national waste management, and infection prevention and control (IPC) strategies and plans.

At the regional level, the Regional Directorates of Health (RDH) are equipped with basic hygiene and sanitation services (BHSS), which are responsible for implementing national guidelines in the country’s six health regions. They ensure coordination between the central and peripheral levels, monitor the implementation of regional plans, and are involved in the supervision of health facilities in terms of hospital hygiene, biomedical waste management and infection prevention (IPC).

The operational level is organized in districts. Each district has a basic sanitation and hygiene service that coordinates the implementation of interventions in biomedical waste management, hospital hygiene, and infection prevention and control (IPC). The districts comprise health units (peripheral units, district hospitals, regional and university hospitals). These structures are responsible for the day-to-day management of biomedical waste. Their performance often depends on the presence of functional waste management committees or PCIs, staff training, and the availability of a budget allocated to this function.

District hospitals, hospitals, regional and university center health units, and some peripheral care units have a hygiene service consisting of hygiene and sanitation technicians. They have technical responsibility for the operational implementation of biomedical waste management activities.

Regulation and planning are the responsibility of management and the various technical committees, including the medical advisory committee, the steering committee, etc., with a proposal from the Hygiene and Sanitation Technician (HST).

Depending on the level of health training, the financing of activities relating to the management of biomedical waste comes from hospital directorates, state resources or the Health Management Committee fund.

The state also receives support from some partners such as WHO, UNICEF, the World Bank, Plan International Togo, AFD, etc.

This research was conducted in public health facilities of all types (type I and II peripheral care units, level I and II district hospitals, regional hospitals and university hospitals), spread over all six health regions and 39 health districts of Togo.

**Figure 1:**
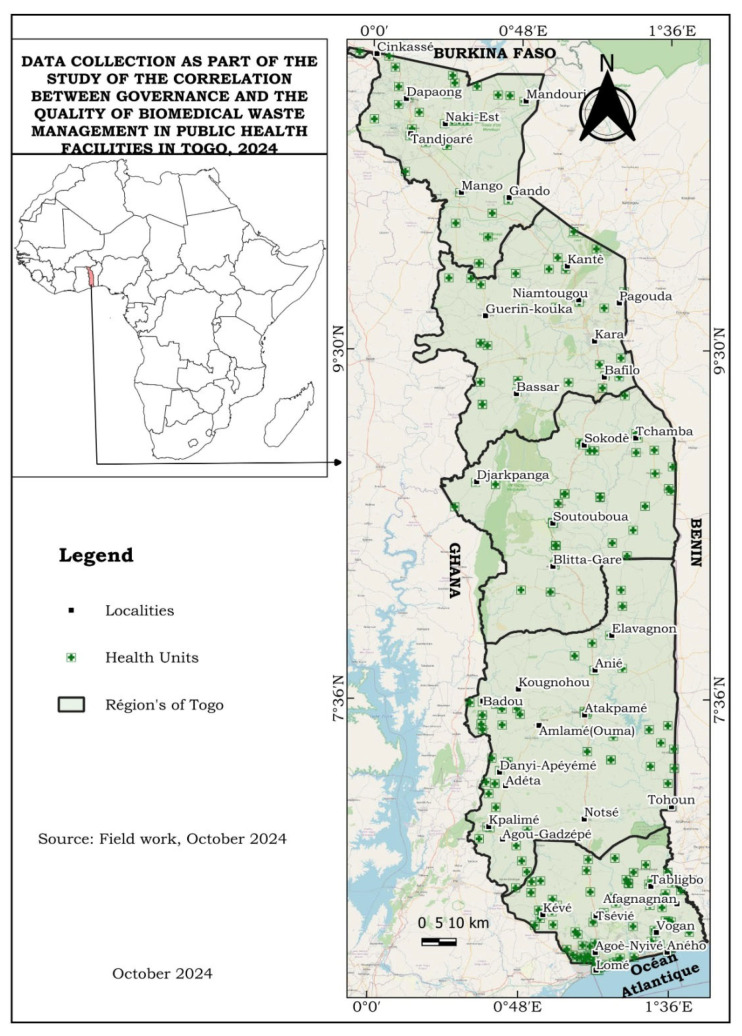
Map of Togo showing the geographical distribution of health facilities surveyed in 2024 (source data collection 2024).

The map of the geographical distribution of the health units surveyed is shown in Figure 1.

### 2.2. Sampling Methods and Techniques

The target groups were the public health units in Togo and healthcare providers and services in the six health regions and 39 health districts of Togo. In order to reduce bias, a probabilistic method with a simple random selection technique was used for the selection of health facilities (HU) to be investigated. The number of HUs of each type relative to the region and district was calculated in proportion to the sample size based on their numerical weight recorded in the official list of public HU of the Ministry of Health by 2022.

For healthcare providers, a non-probabilistic method with the reasoned choice technique was used.

The sample size was determined using Schwartz’s formula:

$$n=\frac{Z^{2}P(1-P)}{e^{2}},$$

where

- n denotes the minimum sample size for each HU level;
- Z denotes the confidence-level fractal;
- p denotes the proportion of each level;
- e denotes the acceptable margin of error in the accuracy of the results.

A total of 264 health facilities were surveyed, and Table 1 shows the distribution of the sample by region.

The distribution of the sample by level of care reflects the national distribution of health units by level of care. Less than one percent of public health institutions (1145) are represented by HUC (3) and RHC (6) at the national level. More than 90% of the national healthcare delivery structures are made up of type 1 and type 2 PCUs.

Type 1 peripheral care unit and type 2 peripheral care unit are the first level of contact and provide basic care (immunization, consultation and delivery) with a low production of biomedical waste.

District hospitals are the primary reference level and provide more advanced care with diagnostic services (laboratory, medical imaging) and sometimes a surgical service.

The second level of reference and recourse is facilitated by regional hospitals (Regional Hospital Centers—RHC). Tertiary healthcare is provided in three University Hospital Centers (HUC) and specialized reference hospitals.

In this way, the quantity and complexity of the waste produced increases with the level of healthcare provision.

Regarding the personnel interviewed in the selected structures, the study targeted actors who hold information on waste management in health facilities. Thus, the study focused particularly on the managers of health facilities, hygiene and sanitation technicians, members of IPC/WASH committees, and technicians in charge of the collection and incineration of biomedical waste. This approach made it possible to target the key informants who are likely to have sufficient and relevant knowledge of biomedical waste management practices within their health training. This hybrid sampling method makes it possible to find a balance between statistical rigor in the choice of health facilities and the specificity of the responses of the actors surveyed.

### 2.3. Variables

The variables used to assess the impact of governance on the quality of biomedical waste management in health facilities were as follows.

#### 2.3.1. Independent Variable

In this research study, governance was the independent variable.

Hospital governance in biomedical waste management refers to regulation; organizational structure; and the planning and funding of the sector for effective, secure, and sustainable management. The governance components of this study were as follows:

- Regulation;
- Planning and financing;
- Organization and training.

Table 2 presents the evaluation indicators via their component.

#### 2.3.2. Dependent Variable

The dependent variable was the quality of biomedical waste management (effective, safe, and sustainable) assessed from the components presented in Table 3.

**Figure 2:**
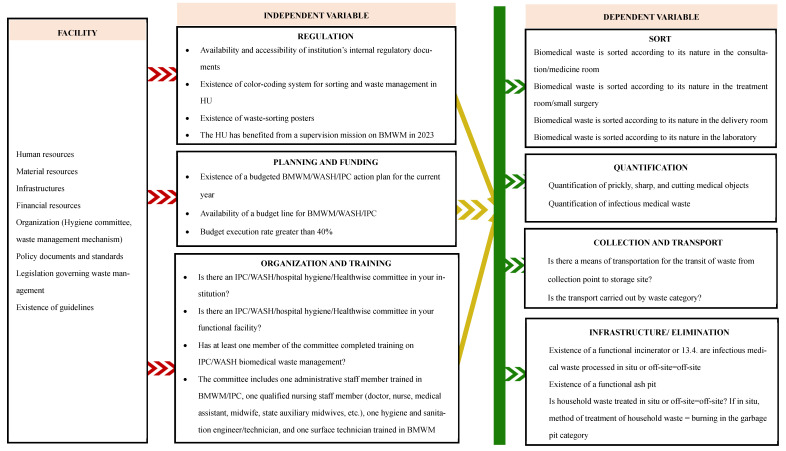
Conceptual framework for the study.

Figure 2 shows the synthesis of the relational logic between the biomedical waste management context, the independent variable, and the dependent variable in our study.

For the purpose of statistical analysis and qualifying performance, Biomedical Waste Management Quality Composite Scores were dichotomized. For instance, health facilities with an overall score of 80% or more were considered as having “adequate” biomedical waste management. This cut-off point is based on the normative assessment methodology suggested by [22], which advocates the use of cut-off points in the assessment of health programs to distinguish between levels of facility performance. It has also been used in analogous assessments of waste management quality and health compliance in resource-poor settings [21,23]. In the case of Togo, the 80% cut-off point was used as a practical and operational choice, in line with the minimum standards detailed in the National Biomedical Waste Management Plan (NPMBW, 2014–2017), and it corresponds to a generally satisfactory level of compliance with basic practices in the segregation, quantification, transportation, and storage facilities of BMW and final disposal.

### 2.4. Organization of Collection

An authorization request was sent to the Ministry of Health for data collection, which was approved. This allowed us to contact the different levels of management in order to present the data collection authorization and explain the purpose of the research study. A collection schedule was established for each region and district. Data collection officers and supervisors were trained, and tools were tested in public health facilities, not including Greater Lomé.

The study involved 24 investigating officers, distributed in the six health regions. They were selected based on minimum qualification criteria in public health or environmental health. These are, in particular, senior public health technicians, state-certified nurses specializing in public health, hygiene and sanitation technicians, and master’s students in public health or hospital management with prior experience in health data collection.

All investigators received three-day training on the research protocol and its objectives, the evaluation grid used, the handling of the digital tool KoboCollect, ethics and confidentiality in conducting interviews, and methods and techniques for observing technical indicators in the field (sorting, infrastructures, displays, documents).

The supervisors involved are engineers and doctors who specialize in public health and sanitation with strong experience in data collection.

To ensure the independence and reliability of the data, all actors involved in the organization and collection of data were identified outside the public health system.

A quality control system has been set up through several stages. Digital validation in real time via KoboCollect, the integration of validation criteria and mandatory fields into the design of the questionnaire.

Supervisors had access to completed electronic records and performed daily verification.

### 2.5. Ethical and Deontological Aspects

The protocol for this research was submitted to the Committee on Bioethics for Health Research (CBHR) of the University of Lomé, which validated it. This validation allowed us to obtain authorizations from the Ministry of Health and Public Hygiene for effective data collection. Meetings were then held with the Regional and Prefectural Directors of Health and Public Hygiene, as well as with the heads of health units (HHUs) to explain the study’s objective, methods, and organization. The health units (HUs) were included after free and informed consent was provided by their HHU, as well as from the providers interviewed. Confidentiality was respected during data collection via measures such as interviewing respondents in an office where they were alone; the data were then stored securely.

### 2.6. Data Processing and Analysis

Data collection was carried out through the application “Kobocollect”. Each submitted form was validated by supervisors, and the data were then exported to SPSS software, version 20 for analysis, which consisted of calculating proportions, means, and standard deviations. For the analysis of relationships between governance indicators and quality of biomedical waste management (BMWM), chi-two tests (χ^2^) were used for bivariate analyses. A *p*-value of less than 0.05 was considered statistically significant. The chi-square test was used to compare proportions, and logistic regression was used to determine relationships between independent variables and the quality of biomedical waste management.

For the logistic regression model, we used the R software, version 4.5. Thus, a binary logistic regression was then used to identify independent associations between the governance components and the quality of the BMWM. Indeed, each criterion of waste management quality (sorting, quantification, collection and transport, infrastructure for elimination) has been separately exposed to all the components of governance (regulation, planning and funding, organization and training) with the aim of analyzing the type of association.

### 2.7. Limitations of the Study

This study has certain methodological limitations. The cross-sectional design made it possible to observe only practices at a specific point in time, without being able to establish a causal link between the dimensions of governance and the quality of biomedical waste management. Another potential bias could be linked to the respondent’s level of technical knowledge concerning biomedical waste management. The level of knowledge about biomedical waste management differs depending on whether the respondent is a doctor, a nurse, a midwife, or a hygiene and sanitation technician. In addition, certain external contextual variables, such as interruptions in the delivery of certain waste management components like safety boxes, are not taken into account. In fact, safety boxes are supplied by public health programs. Thus, a breakdown at a health facility is not necessarily attributable to that facility.

## 3. Results

### 3.1. Description of Health Facilities and Profiles Surveyed

In total, 264 health facilities were covered by the study. The Upper Lomé health units were the most represented, with a proportion of 39.4%, followed by those in the Plateau region at 25.0%.

The data show that Type I and II Peripheral Care Units (USPs) represent 91% of the sample, with 65.2% for I and 25.8% for II. This distribution reflects the pyramid structure of the Togolese health system, where most services are provided at the primary level. This strong representation of first-level structures implies specific challenges in biomedical waste management (BWM), including insufficient resources, lack of technical skills, governance is often poorly structured and the capacity to integrate regulatory and technical requirements is limited due to a lack of continuous training and appropriate materials. In addition, the low level of functionality of waste management committees and trained personnel in these lower-tier facilities exacerbate the risk of poor practices, such as inadequate sorting, lack of incineration or inadequate waste disposal.

Regarding the profiles of the actors surveyed, hygiene and sanitation technicians (HSTs) represented 42.0% of the respondents, followed by state graduate nurses at a proportion of 35,6%. The two categories of professionals were chosen because of their strategic functions at the interface between operations and governance within healthcare facilities.

Hygiene and sanitation technicians have a central position in the daily implementation of biomedical waste management activities. They coordinate waste sorting, collection, internal transport and disposal. They are also responsible for maintaining waste management infrastructures (incinerators, ash pits, storage rooms), preparing waste management action plans and reports. Their involvement in PCI/GDBM committees as active members gives them an integrated view of both technical and strategic issues, making them key informants on biomedical waste management governance.

Nurses are often in charge of peripheral care units, particularly in first-level health facilities. As such, they have a key role to play in planning waste management expenditure and ensuring compliance with hygiene practices within their departments. Their involvement in PCI/GDBM committees reinforces their position in coordinating hygiene activities, raising staff awareness, and monitoring the application of the procedures. Thus, despite being less directly involved in waste handling, their role is essential in ensuring that organizational guidelines are effectively applied within the units.

The predominance of these two profiles among the respondents guarantees the credibility of the data collected, given that they are directly involved in the governance dynamics (implementation, supervision, coordination and evaluation, funding) of biomedical waste management. Collecting data from these two profiles helps to establish solid links between the functional roles of actors and governance mechanisms within health facilities, which is crucial for understanding the institutional determinants of waste management quality.

### 3.2. Governance Indicators Associated with the Quality of Biomedical Waste Management

- Regulation for Biomedical Waste Management

In univariate analysis, all criteria of the regulation studied, namely, the accessibility of internal documents, the existence of waste-sorting posters, the existence of a color-coding system, and supervision, were associated with the quality of biomedical waste management. Similarly, regulation contributes positively and significantly to the effective management of biomedical waste. The analysis shows that all regulatory criteria have an association with the quality of biomedical waste management, with a *p* value below 0.05.

- Organization and Training

Table 4 shows that all organizational and training criteria studied had a statistically significant association with waste management quality. Health units with a Biomedical Waste Management Committee (BMWMC) or an Infection Prevention and Control Committee (IPCC) accounted for 63.3%. Health units with dedicated waste management staff accounted for 62.1% compared to 37.9% without such staff. The administrative staff were not trained in 30.3% of cases, and at least one qualified staff member of the health establishments surveyed received training in biomedical waste management in 68.9% of cases. The analysis shows that all indicators of organization and training show an association with the quality management of biomedical waste, with *p* values below 0.05.

- Planning and Funding

Table 4 shows the planning and financing indicators studied in this study. Public health establishments with a 2024 operational action plan that did not take into account the management of biomedical waste accounted for 42.8%. The planning and funding aspect that shows a link with biomedical waste management quality is (i) the existence of an operational action plan taking into account BMWM/IPC and (ii) a budget line relative to BMWM/IPC, with *p* values below 0.05.

- Illustration of the different types of association

**Figure 3:**
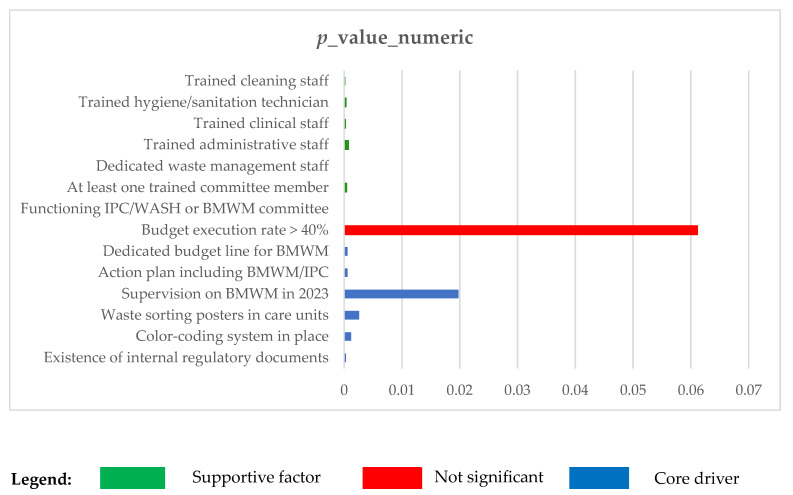
Analysis of *p*-value values.

The results presented in Table 4 show that several governance indicators are statistically associated with biomedical waste management (BMWM) quality. However, they do not all have the same explanatory weight. In order to guide operational recommendations, we distinguish here the “key factors” strongly associated with the performance of “supporting factors”, which strengthen the institutional environment but have an indirect effect. Figure 3, based on the analysis of numerical *p*-values, shows the governance criteria and the different degrees of association with the quality of waste management.

### 3.3. Quality of Biomedical Waste Management

The criteria used to assess the quality of waste management in health training are as follows:

- Sorting;
- Quantification;
- Collection and transport;
- Infrastructure for elimination;
- Observation of the environment.

The multivariate analysis makes it possible to assess the significance criteria. Table 5 presents the results of the multivariate analysis.

Table 5 shows that the significant criteria are sorting, quantification, transport, and elimination infrastructure. The application of this grid shows that 17.8% of the HU surveyed had a score greater than or equal to 80% and therefore managed waste in an “acceptable” manner, as shown in Table 5. This table also shows that the management quality of BMW was unevenly distributed among the types of HU surveyed. This result highlights the critical steps to ensure the safe and environmentally sound management of biomedical waste.

Most of these high-performance facilities are located in urban areas, with a predominance in the capital of the Grand Lomé health region and its outskirts (46.8%). In terms of healthcare provision, these are essentially regional hospital centers (UHC), university hospital centers (UHC) and district hospitals. There are, however, a few exceptions: well-equipped peripheral facilities in the Plateaux and Maritime regions, and district hospitals generally located in the main towns of the districts.

These high-performance health facilities have a good internal governance and adequate infrastructure in common. Almost all high-performance facilities have an active ICP committee, staff trained in BMW at several levels (nursing, administrative, technical), and a dedicated waste management officer. Hospitals also have an incinerator in working order, space before storage and logistical resources for transporting waste, enabling them to follow technical procedures.

### 3.4. Association Between Governance and Management Quality of BMW

A multivariate analysis with the logistic regression model consisted of evaluating the strength of the association between the modalities of the independent variable and those of the dependent variable.

- Waste management sorting and good governance

The analysis of results in Table 6 shows that organization-specific governance and training contribute to improved sorting (OR = 1.029; 95% CI [1.01; 1.047]), which is an important criterion for the quality of biomedical waste management.

- Quantification and good governance of waste management

The analysis of the results in Table 6 shows that specific organizational and training governance contributes to the effective and regular implementation of the BMW quantification operation (OR = 1.037; 95% CI [1.018; 1.057]), which is an important criterion in the forecasting and planning of the installation of biomedical waste equipment and infrastructure.

- Collection and transport and good waste management governance

The analysis of the results in Table 6 shows that organization-specific governance and training contribute to the improved collection and transportation of BMW (OR = 1.046; 95% CI [1.026; 1.066]), which is an important step in the secure management of BMW.

- Infrastructure for elimination and governance.

The analysis of the results in Table 6 shows that governance specifically focuses on organization and training for the establishment and maintenance of waste disposal infrastructure (OR = 1.037; 95% CI [1.018; 1.056]). The organization understands the establishment and operation of BMWM/IPC committees and the existence of an operational action plan (OAP) for BMWM/IPC. The existence of a functional committee and an OAP are factors favoring the construction and maintenance of BMW disposal infrastructures (incinerator, ash pit, etc.).

**Figure 4:**
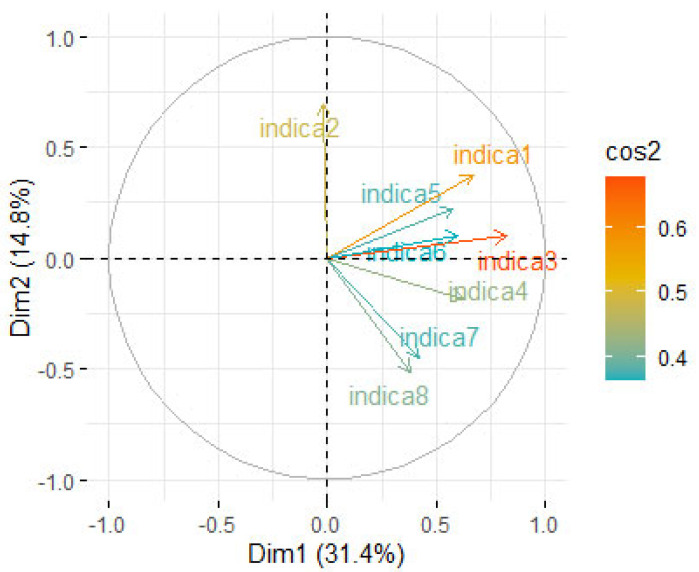
Correlation circle of variables.

**Figure 5:**
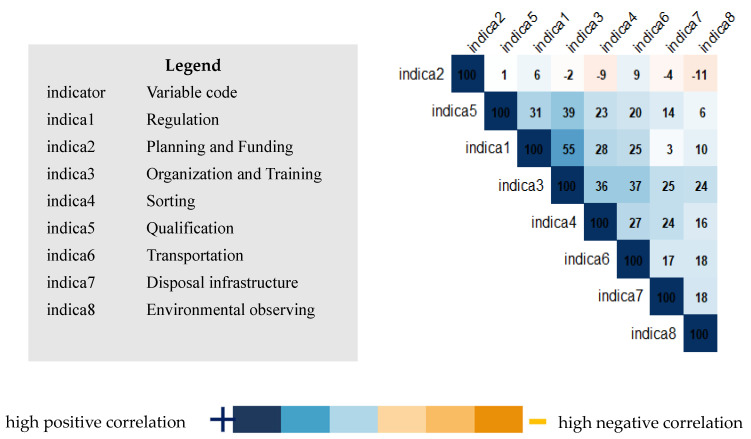
Correlation matrix of variables.

The analysis of Figure 4 and Figure 5, representing, respectively, the correlation circle of variables and the correlation matrix of variables, clearly confirms the significant relationship between the “Organization and Training” component (Indicator 3) and the biomedical waste management quality indicators, specifically sorting (Indicator 4), quantification (Indicator 5), transportation (Indicator 6), disposal infrastructure (Indicator 7), and the environmental conditions surrounding health facilities (Indicator 8). This correlation underscores the importance of staff training and internal organizational structures as key factors for effectively improving biomedical waste management in the surveyed health facilities.

Following the cross-reference between the various quality criteria and governance components, Table 7 presents the results of the cross-reference model between all quality components, including sorting, quantification, transport, and availability of disposal infrastructures with significant governance components, including regulation and “organization and training”. The analysis confirms the results of the previous tables. Governance, including organization and training, contributes significantly to improving the quality of waste management in health facilities (OR = 3.7966; 95% CI [1.7940; 8.0347]).

Multivariate analysis using logistic regression revealed that governance, notably through staff training and internal organization, is the key determinant associated with biomedical waste management (BMWM) performance. A detailed examination shows that among the organization and training variables, the presence of a functional PCI/WASH or BMWM committee is associated with an Odds Ratio (OR) of 3.79 (95% CI: 1.91–7.50). This means that a facility with such a committee is almost four times more likely to meet or exceed the BMWM quality threshold (≥80% compliance), compared with a facility without an active committee. Similarly, the presence of trained staff at different levels (nursing, hygienists, administrative) and the assignment of a dedicated waste management officer are also statistically demonstrated performance levers, all associated with ORs greater than 2.

Thus, the study reveals that the establishment and operationalization of IPC/BMWM committees in all health facilities and the systematic institutionalization of initial and ongoing training on waste management in all health training structures are key operational pillars for sustainable improvement in biomedical waste management in health facilities. These are moderate-cost, high-impact levers that can be activated in the short term.

## 4. Discussion

The general objective of our study was to analyze the correlation between governance in public health establishments in Togo and the quality of biomedical waste management within them. In this study, we included 264 public HUs of all types, using the simple random sampling technique via strata to minimize selection biases.

- Most critical steps in the biomedical waste management circuit

The data analysis showed that among the criteria for assessing the quality of biomedical waste management (BMWM), sorting is the most significant (OR = 1.482; 95% CI [1.286; 1.708]), followed by quantification (OR = 2.026; 95% CI [1.491; 2.753]), transportation (OR = 1.403; 95% CI [1.187; 1.66]), and elimination infrastructure (OR = 1.604; 95% CI [1.298; 1.982]). The application of the grid shows that 17.8% of the surveyed HU had a score greater than or equal to 80% on all criteria used for assessing the quality of management of BMW and therefore managed waste in an “acceptable” manner.

Biomedical waste management is qualified as “good” in health training when the health training has a score of at least 80% on all components of the dependent variable that defines the quality criteria for biomedical waste management, including sorting, quantification, collection and transport, and disposal infrastructure.

Indeed, triage is effective in 59.1% and 53.8%, respectively, of the consultation and delivery services of health facilities covered in the study.

Sorting is essential to ensure the safe and environmentally sound management of biomedical waste. It allows for their categorization, limits hazardous waste, and promotes their recovery. Sorting at source reduces the amount of infectious waste by avoiding contamination of non-infectious waste, thus reducing management costs and limiting pollution from the incineration of biomedical waste. Sorting helps reduce the risk of blood exposure accidents (BEA) associated with waste handling.

However, this stage encounters many difficulties in the health services of developing countries, as reported by [13] in a study on biomedical waste management in the public healthcare structures of district 4 in Lomé [24], as well as [20] in Côte d’Ivoire. Our study found that only 59.1% and 53.8% of the consultation and delivery services practiced sorting, which is a proportion close to the 55% reported in the evaluation of the National Plan of Biomedical Waste Management (NPBMWM) 2014–2017 in 2020 [4,13]. In a study carried out by Adon, it emerged that waste sorting was neither systematic nor widespread in the health facilities of Abidjan [20]. However, for [19], in Cameroon, biomedical waste sorting was systematic in only 46.5% of the health facilities included [19,21,24]. Similarly, 76% of the providers surveyed had acknowledged that source sorting was not systematic in a study conducted by Landry et al. at the Kitambo Reference Hospital in the Democratic Republic of the Congo in 2024 [18,21,25]. In a study on the knowledge and perceptions of healthcare providers relative to the environmental effects of biomedical waste at university hospital centers (UHCs) in Togo [25] and [23] in a study carried out in Côte d’Ivoire [1,25]. The difference in these proportions could be explained by the time elapsed between these studies and ours: 12 years and 4 years for the first two. In addition, these studies have been carried out in other countries with realities that may differ from those of Togo. They were carried out with respect to a limited number of structures, while ours involved 264 units of care, including all regions, districts, and types of health facilities in Togo [21].

In this study, a number of factors explain this situation, including insufficient training for those involved, the unavailability of waste garbage cans and bags, the absence or low effectiveness of monitoring and supervision, the absence of procedures and posters on waste sorting, and the low level of knowledge of the risks associated with biomedical waste. This finding is the same as that of [26], who came to the same conclusion that waste sorting was hampered by the absence of coded containers, lack of training and inadequate supervision.

Sorting is facilitated via color coding or the chromocoding that is intrinsically related to it. In our study, the color-coding system existed only in 65.1% of the surveyed structures. Color coding is not used in 34.9% of the facilities surveyed, which compromises the efficiency of sorting in these facilities. Color coding is used to label containers according to the type of waste they are supposed to contain. This is the basis for efficient sorting of biomedical waste at source. In their study, [19] found that color coding was non-existent. However, Ndiaye, in the study mentioned above, noted that color coding was used only in 31.4% of all services surveyed. Periodic reviews of the different levels of responsibilities of the providers in various structures can be used as an argument to explain the difference between these proportions and ours. These types of oversights are, according to [24].

Insufficient sorting in health facilities results in environmental pollution, the misuse of resources, and increased costs for medical facilities [11]. The lack of sorting results in an increase in the quantity of waste with infectious risks and therefore increases in the costs of treatment, environmental impact, and public health risks.

The quantification of biomedical waste generated is a practice that aims to make information on waste generated by different categories available. It also provides resources for the proper management of these waste materials. However, the quantification was carried out relative to only a small proportion of structures (31.1% for prickly, sharp, and cutting medical objects and 23.9% for infectious medical waste), reported a lack of reliable data on the quantities of waste generated [21,23]. This may also be explained by the lack of a standardized institutional system for quantification and the lack of other tools intended solely for quantification. Indeed, less than 25% of health facilities have a register for the registration of infectious medical waste quantities and prickly and sharp medical objects. Waste quantification allows for better forecasting of waste quantities and waste disposal systems planning. The lack of quantification does not allow a good forecast and the adequate dimensioning of biomedical waste treatment infrastructures at the level of health training and the national level, further preventing the adequate positioning of incinerators.

Safe transportation prevents waste accumulation and reduces the risk of injury [27]. The study found that 38% do not transport biomedical waste safely. This situation is explained by the lack of adequate means of transport; transport is carried out with wheelbarrows at worst by bare-handed workers without personal protective equipment (PPE). The structures do not have operational procedures or a standardized waste transport circuit, sometimes the transport is not carried out separately leading to contamination risks. Analysis of available action plans shows that the planning does not include a budget for the funding of biomedical waste management logistics.

The proportion of HU with incinerators comprised 57.9% and 8.5% of available incinerators that were not functional. Indeed, the study revealed an insufficiency in the preventive and curative maintenance of both Montfort and conventional-type incinerators. The low-level functionality of incinerators is related to the misuse of mis-sized refractory bricks that have not reached 800 °C. This situation is also linked to the insufficient planning and insufficient consideration of investments related to the construction and maintenance of waste management infrastructures in the budget for health facilities. Indeed, incinerators are almost 100% financed and rehabilitated by projects, which explains the low coverage rate.

Poorly managed incinerators produce dioxins and furans, which are carcinogenic cyclic compounds with consequences for public health and the environment. Emissions from the incinerator contribute significantly to polychlorinated dibenzo-p-dioxins (PCDD), polychlorinated dibenzofurans (PCDF), and dioxin-like polychlorinated biphenyls (PCB) according to various studies [28]. It is therefore important to use less expensive and polluting methods such as sterilizers.

- Association between governance and management quality of BMWM

The multivariate analysis showed a statistically significant association between the organization and training component and the quality of biomedical waste management (BMWM) in health facilities (OR = 3.79; 95% CI [1.79–8.03]; *p* < 0.001). For example, establishments with functional waste management committees, personnel trained in the management of biomedical waste, and dedicated officers have a nearly four times higher probability (OR = 3.79) of good performance.

This is in line with the findings of [25], who emphasized the importance of local coaching and continuing education as drivers of waste management quality.

The main criteria of the organization and training components are the existence of qualified personnel trained in BMWM in recovered health facilities, the functionality of a BMWM/IPC committee, and the presence of at least one qualified member of staff trained in DBM management.

Governance functions as a mechanism for the accountability of actors at all levels and across services through the interventions of the IPC/BMWM committee which plans, organizes and provides day-to-day oversight of waste management activities.

The functionality of a BMWM/IPC committee is an important criterion for continuous improvement in biomedical waste management in health education. Indeed, this multidisciplinary committee (management, finance, hygiene service, providers, etc.) is a reference framework par excellence for analysis, identification of solution approaches, and, especially, for the planning of interventions and resources for strengthening biomedical waste management. For example, more than 75% of the HUs with GDBM/PCI committees had a PAO for biomedical waste management. More than 80% of the MSDSs with a committee and DSO had “satisfactory” management of BMRs. Ref. [21], in his study “Evaluation of the management of biomedical solid and liquid waste in university hospitals (UHC) in Togo in 2021”, has also demonstrated a significant association between the poor management of BMRs and the absence of a BMWM/IPC committee, insufficient training of healthcare providers and lack of a operational action plan for biomedical waste management.

The training strengthens stakeholders’ capacity to understand the risks associated with waste management while providing them with best practices and procedures in accordance with national and international standards [8]. Health facilities with at least one qualified healthcare provider trained in BMWM accounted for 68.9%, a far cry from the 33% found by Landry in the DRC [17]. In fact, in his study, Landry assessed the number of providers trained in BMWM while our study sought to know if the structure had at least one qualified healthcare provider.

Thus, the presence of a functional committee or trained staff is a key factor in improving biomedical waste management in health training.

- Weak link to regulation and funding

The regulatory and planning/financing components were not significantly associated with the quality of BMWM in multivariate analysis, although they exhibited a favorable trend in bivariate analysis. This result could reflect a gap between the existence of texts or budget lines and their effective operationalization in health facilities. It is noted that regulations, trained technical resources, and a functional BMWM/IPC committee exhibit low impacts. This corroborates the findings of [19] with respect to the ineffectiveness of unenforced regulatory frameworks.

Our study contributes to the understanding of the action of governance on the quality of waste management and allows us to determine—in a context of limited resources such as that of developing countries—the relevant elements of governance that can be emphasized to improve management.

Overall, our study confirms that training and organization are positively associated with good biomedical waste management. However, the majority of public health establishments (62.8%) in Togo were mismanaging biomedical waste. This confirms most of the studies carried out in this area, which highlight unsatisfactory management of biomedical waste with respect to health structures. Indeed, in 2002, a WHO study in 22 developing countries found that up to 64% of care facilities did not properly apply appropriate waste disposal methods. This situation could be explained by budgetary constraints marked by multiple pressing needs, resulting in a lack of financing for waste management. The other explanation may lie in the fact that the national regulation on BWM is not well known by service providers and administrators; moreover, the structures themselves do not have internal waste management legislation, and this rarely instills users with awareness of these issues.

Despite the absence of a significant link in the multivariate analysis between the “planning and financing” component, its role in the sustainable improvement of biomedical waste management remains structurally crucial. Indeed, several studies point out that the lack of a specific budget for biomedical waste management is a major obstacle to the implementation of compliant practices, even in the presence of adequate regulations or training [2]. It is the financial availability that allows the acquisition of inputs (garbage cans, garbage bags, fuels, etc.) for waste management.

Our analysis shows that more than 42% of the establishments did not have an operational action plan including biomedical waste management, and 51.5% did not have a specific budget envelope. This may be explained by the weak integration of biomedical waste management into institutional planning and the lack of financial empowerment of institutions. As pointed out by [23], the chronic underfunding of environmental health in sub-Saharan Africa limits access to functional equipment (incinerators, sorting tanks), but also the recruitment and ongoing training of dedicated staff.

This discrepancy between the theoretical planning (existence of budget lines) and the effectiveness of actions may also reflect a low execution rate of voted budgets. Thus, despite the lack of a strong link at the statistical level, the results suggest that strengthening budgetary planning, Monitoring the implementation of credits and integrating the BMWM into health services development plans are strategic levers to sustainably improve the management of biomedical waste in public facilities.

To ensure a sustainable improvement of the situation, waste management performance, such as the existence of a functional ICP/BMWM committee, must be taken into consideration in the evaluation criteria for health facilities, and its performance must also be linked to the accreditation and financing of health facilities.

- Practical and immediate implications of this study for policy makers

This study highlights the importance of governance in improving biomedical waste management. It identifies precisely the levers that can be activated in the short term and at low cost for a sustainable improvement of biomedical waste management in health facilities.

These results lead us to proposals for concrete measures at the operational level (health training) and strategy at the national level.

Thus, at the level of health establishments, it will be necessary to set up and make operational the PCI/GDBM committees, to adopt the guide on national biomedical waste management, to organize weekly meetings for follow-up of the production of biomedical waste in the different departments with the help of evaluation grids. Performance by service must be monitored weekly at management committee meetings or medical advisory board (MAB) meetings. Finally, to support this approach in the long term, it is important to integrate biomedical waste management activities into the operational action plan of health facilities with budget lines.

At the national level, emphasis will be placed on the following aspects, including the establishment of a mapping of stakeholders involved in bio-medical waste management, the development of a national biomedical waste management plan, the planning and implementation of monitoring at all levels (national, regional, and district), advocacy for the establishment of a budget line at the national level for biomedical waste management, integration of waste management modules in the training curricula of all health training structures. In order to strengthen monitoring, it will be important to implement digital tools for monitoring the effectiveness of waste management in health drilling. The national level should also develop and disseminate protocols and procedures in accordance with national regulations and international standards. In order to sustain all its interventions, it is recommended that an interdepartmental working group on biomedical waste management be established.

## 5. Conclusions

This study focused on the correlation between the governance of public health establishments in Togo and the quality of biomedical waste management.

The results showed clearly that only the organizational and training dimensions had a statistically significant association with quality biomedical waste management. This finding supports the hypothesis that internal governance mechanisms, such as staff training and the establishment of functional committees, have a more decisive effect than non-operational regulatory or financial arrangements.

This finding reinforces the relevance of targeted interventions at facility level, focusing on strengthening human capacities, empowering local players, and structuring biomedical waste management practices internally.

Based on these results, the study recommends that at the operational level, short-term actions such as staff training, the establishment and operationalization of IPC/BMWM committees, or the integration of waste management activities into action plans are both inexpensive and easily applicable. At a national level, the study recommends the establishment of a mapping of stakeholders involved in biomedical waste management, the development of a national biomedical waste management plan, the planning and implementation of monitoring at all levels (national, regional and district), and the integration of waste management modules in the training curricula of all health training structures. Finally, to ensure the sustainable improvement of the situation, waste management performance, such as the existence of a functional ICP/BMWM committee, must be taken into consideration in the evaluation criteria for health facilities, and its performance must also be linked to the accreditation and financing of health facilities.

In addition, the heterogeneity of results depending on the type of health training facility calls for an in-depth analysis of specific contexts: human resources, managerial capacities, or the local institutional environment. A follow-up study, incorporating these contextual variables, would enable us to develop finer-tuned, better-targeted interventions.

Although focused on the Togolese context, this research highlights issues common to many low- and middle-income countries facing similar challenges in hospital governance and sustainable management of medical waste. The analytical model proposed here can thus be adapted or tested in other contexts, helping to enrich international thinking on sustainable MRW management systems in resource-limited environments.

## Figures and Tables

**Table 1:** Distribution of sample size by the type of health unit and region.

	Type of Health Unit to be Investigated						Total
	HUC ^6^	PCU II ^1^	PCU I ^2^	RHC ^5^	DH I ^3^	DH II ^4^	
Health region							
Upper Lomé	1	37	51	1	11	3	104
Maritime	0	8	34	0	0	1	43
Plateaux	0	14	47	0	2	3	66
Centrale	0	2	11	0	0	0	13
Kara	0	5	18	0	1	0	24
Savanes	0	2	11	0	1	0	14
Total	1	68	172	1	15	7	264
Percentage (%)	0.38	25.76	65.15	0.38	5.68	2.65	100.00

^1^: Type 1 Peripheral care unit; ^2^: type 2 peripheral care unit; ^3^: level I district hospital; ^4^: level II district hospital; ^5^: regional hospital center; ^6^: university hospital center.

**Table 2:** Indicators selected via the governance component for the study of the correlation between the governance and quality of BMWM in the public HU of Togo in 2024.

Modality	Indicator	Quotation	Rate
**Regulation**	Availability and accessibility of institution’s internal regulatory documents	1	27%
	Existence of color-coding system for sorting and waste management in HU	1	
	Existence of waste-sorting posters	1	
	The HU ^1^ has benefited from a supervision mission on BMWM ^2^ in 2023	1	
**Total Regulation**		4	
**Planning and funding**	Existence of a budgeted BMWM/WASH/IPC ^3^ action plan for the current year	1	20%
	Availability of a budget line for BMWM/WASH/IPC	1	
	Budget execution rate greater than 40%	1	
**Total Planning and funding**		3	
**Organization and training**	Is there an IPC/WASH ^4^/hospital hygiene/Healthwise committee in your institution	1	53%
	Is there an IPC/WASH/hospital hygiene/Healthwise committee in your functional facility	1	
	Has at least one member of the committee completed training on IPC/WASH biomedical waste management	1	
	Existence of at least one staff member dedicated to waste handling and incineration	1	
	Existence of at least one administrative staff member trained in BMWM/IPC after January 2020	1	
	Existence of at least one qualified nursing staff member (doctor, nurse, medical assistant, midwife, state auxiliary midwives, etc.) trained in BMWM/IPC after January 2020	1	
	Existence of at least one hygiene and sanitation engineer/technician trained in BMWM/IPC after January 2020	1	
	Existence of at least one surface technician trained in BMWM/IPC after January 2020	1	
**Total organization**		**8**	

^1^ Health Unit, ^2^ Biomedical Waste Management, ^3^ Infection Prevention and Control, ^4^ WASH: Water, Sanitation, and Hygiene.

**Table 3:** Indicators selected by components of biomedical waste management quality for the study of the correlation between the governance and quality of BMWM in Togo’s public health services in 2024.

Modality	Indicators	Quotation	%
**Sort**	Biomedical waste is sorted according to its nature in the consultation/medicine room	1	
	Biomedical waste is sorted according to its nature in the treatment room/small surgery	1	
	Biomedical waste is sorted according to its nature in the delivery room	1	
	Biomedical waste is sorted according to its nature in the laboratory	1	
**Total Sort**		4	29%
**Quantification**	Quantification of prickly, sharp, and cutting medical objects	1	
	Quantification of infectious medical waste	1	
**Total Quantification**		2	14%
**Collection and Transport**	Is there a means of transportation for the transit of waste from collection point to storage site	1	
	Is the transport carried out by waste category	1	
	Is there a storage room?	1	
**Total Transport**		3	21%
**Infrastructure/Elimination**	Existence of a functional incinerator or 13.4. are infectious medical waste processed in situ or off-site = off-site	1	
	Existence of a functional ash pit	1	
	Are household waste treated in situ or off-site = off-site. If in situ, method of treatment of household waste = burning in the garbage pit	1	
**Total Infra/Elimination**		3	21%
**Observation of the HU environment**	Are there any traces of prickly, sharp, and cutting medical objects (syringes and lancets) in the immediate environment of health training = no	1	
	Are there any infectious medical waste (tampons, blood-stained sparadrap) in the immediate environment of the health training = no	1	
**Total Observation**		2	14%

**Table 4:** Governance indicators associated with the quality of biomedical waste management.

Government Components	Criteria	Management Quality No (n (%))	Management Quality Yes (n (%))	Total (N (%))	*p*-Value
**Distribution of health facilities surveyed according to regulatory criteria**	Accessibility of internal documents	151 (69.6)	19 (40.4)	170 (64.4)	**0.0003**
	Existence of a color-coding system	84 (38.7)	6 (12.8)	90 (34.1)	**0.0012**
	Existence of waste sorting posters	120 (55.3)	14 (29.8)	134 (50.8)	**0.0026**
	Supervision of waste management	108 (49.8)	14 (29.8)	122 (46.2)	**0.0198**
	Regulation (good)	34 (15.7)	16 (34.0)	50 (18.9)	**0.0067**
**Distribution of health facilities surveyed according to organization and training criteria**	Existence of a BMWM/IPC/WASH committee	92 (42.4)	5 (10.6)	97 (36.7)	**<0.0001**
	Functional committee (n = 167)	57 (45.6)	11 (26.2)	68 (40.7)	**0.0419**
	Presence of at least one trained member	121 (55.8)	8 (17.0)	129 (48.9)	**<0.0001**
	Dedicated waste management staff	96 (44.2)	4 (8.5)	100 (37.9)	**<0.0001**
	Surface technician trained in BMWM	166 (76.5)	24 (51.1)	190 (72.0)	**0.0008**
	Administrative staff trained in BMWM	161 (74.2)	23 (48.9)	184 (69.7)	**0.0012**
	Hygiene technician trained	169 (77.9)	20 (42.6)	189 (71.6)	**<0.0001**
	Qualified healthcare staff trained in BMWM	78 (35.9)	4 (8.5)	82 (31.1)	**0.0004**
	Organization and training (good)	26 (12.0)	18 (38.3)	44 (16.7)	**<0.0001**
**Distribution of surveyed health facilities relative to planning and financing criteria**	Existence of an operational action plan including BMWM/IPC	104 (47.9)	9 (19.2)	113 (42.8)	**0.0006**
	Existence of a budget line for BMWM/IPC	123 (59.4)	13 (31.7)	136 (51.5)	**0.0006**
	Acceptable execution rate (>40%)	23 (27.4)	8 (28.6)	31 (27.7)	**1.0000**
	**Planning and funding (good)**	**61 (28.1)**	**20 (42.5)**	**81 (30.7)**	**0.0764**

IPC/WASH: Infection prevention and control/water, sanitation, and hygiene; BMWM: Biomedical Waste Management. Infection Prevention and Control.

**Table 5:** Most critical steps in the biomedical waste management circuit.

Efficient Waste Management	Odds Ratio	t-Value	*p*-Value	[95% Conf Interval]		Sig
**Sorting**	1.482	5.42	0	1.286	1.708	***
**Quantification**	2.026	4.51	0	1.491	2.753	***
**Collection and transport**	1.403	3.96	0	1.187	1.66	***
**Infrastructure for elimination**	1.604	4.38	0	1.298	1.982	***
**Observation of the Environment**	1.087	1.10	0.269	0.937	1.261	

Note: *** show significant criteria.

**Table 6:** Waste management sorting and good governance.

Management Quality of BMW	Governance Components	Odds Ratio	*p*-Value	[95% Conf Interval]		Sig	Nature of the Correlation
**Sorting**	Regulation	9.432	0.176	0.365	243.506	No	
	Planning and Funding	0.934	0.063	0.869	1.004	No	
	Organization and Training	1.029	0.002	1.01	1.047	Yes	High positive correlation
**Quantification**	Regulation	20.341	0.079	0.704	58.427	No	
	Planning and Funding	1.014	0.718	0.94	1.093	No	
	Organization and Training	1.037	0	1.018	1.057	Yes	High positive correlation
**Transportation**	Regulation	2.009	0.679	0.074	54.651	**No**	
	Planning and Funding	1.048	0.211	0.974	1.128	**No**	
	Organization and Training	1.046	0	1.026	1.066	Yes	High positive correlation
**Disposal infrastructure**	Regulation	0.074	0.124	0.003	2.032	No	
	Planning and Funding	0.995	0.892	0.927	1.068	No	
	Organization and Training	1.037	0	1.018	1.056	Yes	High positive correlation

**Table 7:** Logistic regression of indicator groups associated with biomedical waste management quality.

	Odds Ratio	95% Confidence Interval		Coefficient	S.E.	Z-Statistic	*p*	Nature of the Correlation
Regulation	1.9547	0.9138	4.1811	0.6702	0.3879	1.7277	0.0840	High positive correlation
Organization and training	3.7966	1.7940	8.0347	1.3341	0.3825	3.4879	0.0005	High positive correlation

S.E.: Standard error.

## Data Availability

Informed consent was obtained from all subjects.
